# Interchromosomal translocation in neural progenitor cells exposed to L1 retrotransposition

**DOI:** 10.1590/1678-4685-GMB-2022-0268

**Published:** 2023-01-30

**Authors:** Alysson R. Muotri

**Affiliations:** 1 University of California San Diego, Department of Pediatrics, La Jolla, CA, USA.; 2 University of California San Diego, Department of Cellular & Molecular Medicine, La Jolla, CA , USA.; 3 University of California San Diego, Center for Academic Research and Training in Anthropogeny, Kavli Institute for Brain and Mind, Archealization Center, La Jolla, CA , USA.

**Keywords:** L1 retrotransposition, neural progenitor cells, translocations, brain mosaicism

## Abstract

LINE-1 (L1) elements are a class of transposons, comprising approximately 19% and 21% of the mouse and human genomes, respectively. L1 retrotransposons can reverse transcribe their own RNA sequence into a *de novo* DNA copy integrated into a new genomic location. This activity, known as retrotransposition, may induce genomic alterations, such as insertions and deletions. Interestingly, L1s can retrotranspose and generate more *de novo* L1 copies in brains than in other somatic tissues. Here, we describe for the first time interchromosomal translocation triggered by ectopic L1 retrotransposition in neural progenitor cells. Such an observation adds to the studies in neurological and psychiatric diseases that exhibited variation in L1 activity between diseased brains compared with controls, suggesting that L1 activity could be detrimental when de-regulated.

About 20% of the human, rat and mouse genomes are composed of LINE-1 (L1) retrotransposons (Lander et al., 2001; Waterston et al., 2002; Gibbs et al., 2004). Although most L1 elements are retrotransposition defective (Grimaldi et al., 1984; Moran and Gilbert, 2002), the average human genome is estimated to harbor around 100 active L1 elements, whereas the rat contains roughly 400 and the mouse genome around 3,000 active L1 elements (DeBerardinis et al., 1998; Goodier et al., 2001; Brouha et al., 2003; Penzkofer et al., 2005). Active L1 retrotransposons can affect the genome in various ways, creating insertions, deletions, processed pseudogenes, new splice sites, or fine-tuning gene expression, revealing incredible flexibility for genetic manipulation upon integration (Kazazian, 1998; Esnault et al., 2000; Wei et al., 2001; Dewannieux et al., 2003; Perepelitsa-Belancio and Deininger, 2003; Han et al., 2004). Human L1s structurally resemble those present in rodent genomes, and the advent of a cultured cell retrotransposition assay has revealed that human L1s can retrotranspose in a variety of mammalian cell lines (Moran *et al.*, 1996; Wei *et al.*, 2000; Morrish et al., 2002; Han and Boeke, 2004; Muotri, 2016; Macia et al., 2017).

Previous studies have indicated that L1 retrotransposition can occur in germ cells or in early embryogenesis before the germ line becomes a distinct lineage (Ostertag et al., 2002; Prak et al., 2003; Garcia-Perez et al., 2007), and cultured cell retrotransposition assay has revealed that human and mouse L1 elements can retrotranspose in a variety of transformed or immortalized cultured cell lines (Moran et al., 1996; Morrish et al., 2002; Han et al., 2004). Previous data showed that neuronal progenitor cells (NPCs) support L1 retrotransposition during neuronal differentiation *in vitro* and *in vivo*, resulting in a L1 genetic mosaic nervous system (Muotri et al., 2005; Muotri, 2016; Macia et al., 2017). In NPCs in culture, L1 retrotransposons can insert near to or within neuron-associated genes, such as olfactory receptors, ion channel-associated genes, and cadherin receptors. An L1 insertion in the promoter region of the *Psd-93* gene, encoding a post-synaptic density protein involved in different aspects of synapse formation (Conroy et al., 2003; Tao *et al.*, 2003; Parker et al., 2004), significantly increased gene expression level and, consequently, accelerated neuronal maturation in culture (Muotri *et al.*, 2005). An analysis of the sequence data from several L1 insertions in NPCs indicated that the integration process might be regulated and probably targeted open chromatin regions, but size of the target genes might also be a factor (Thomas et al., 2012). It is challenging to determine or precisely predict the consequences of L1 endogenous retrotransposition in neurons. Virtually any RNA molecule can be subject to *in trans* retrotransposition if hijacked by L1 machinery (Esnault et al., 2000; Wei et al., 2001; Kajikawa and Okada, 2002; Dewannieux et al., 2003). Thus, each developing neuron can potentially carry several L1-mediated events, and if some of the resulting insertions occur in genes expressed during neuronal development, it is possible that neuronal networks during brain development could be significantly impacted by *de novo* L1 insertions (Muotri and Gage, 2006; Cao et al., 2006; Baillie et al., 2011; Evrony et al., 2012; Jacob-Hirsch et al., 2018; Shpyleva et al.,2018; Moszczynska, 2020; Bundo and Iwamoto, 2023). The characterization of L1 insertions in NPCs reveals if the neural intracellular milieu can interfere with L1 insertion, helping to pave the way for a better understanding of the role of L1 retrotransposition in the nervous system (Thomas *et al.*,2012; Suarez et al., 2018).

Here, we describe the analysis of an interchromosomal translocation potentially triggered by ectopic L1 retrotransposition in an NPC clone derived from stable rat neural stem cells in culture (HCN). HCN cells represent a heterogeneous population composed of neural stem cells and more differentiated NPCs. An episomal retrotransposition-competent human active L1 element (LRE3) controlled by its endogenous promoter and carried an EGFP reporter construct was used to obtain the Clone C6+ (Muotri et al., 2005). Briefly, the EGFP gene is interrupted by the g-globin IVS2 intron in the same transcriptional orientation as the L1 transcript. This arrangement ensures that EGFP-positive cells will arise only when a transcript initiated from the promoter driving L1 expression is spliced, reverse transcribed, and integrated into chromosomal DNA, thereby allowing expression of the EGFP gene from the pCMV promoter (Moran et al., 1996; Ostertag et al., 2000) (Figure 1a). Cells harboring the L1 expression constructs were selected by the addition of puromycin to the culture medium, and puromycin-resistant cells were screened for EGFP expression by flow cytometry, as previously described (Muotri *et al.*, 2005). The clone C6+ comes from a single EGFP-positive puromycin-resistant cell that was collected by fluorescence-activated cell sorting (FACS) 7 days post-electroporation (Figure 1b). As previously reported, the EGFP expression was reduced over time due to epigenetic modifications at the L1-EGFP insertion site (Muotri *et al.*, 2005). Sequencing of the PCR product from the clone C6+ confirmed that the intron in the EGFP gene was spliced precisely (Figure 1c).

**Figure 1 -:**
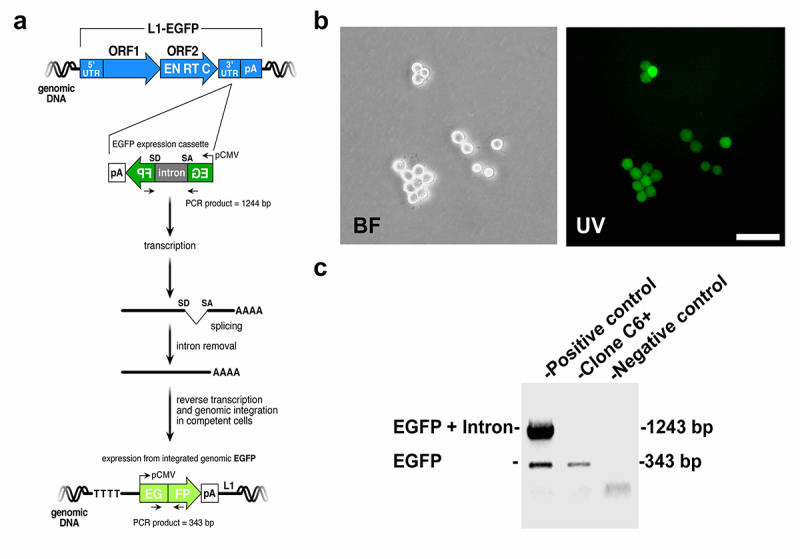
Isolation of clone C6+ by L1-EGFP retrotransposition. **a**, The retrotransposition-competent human L1 (L1_RP_) contains a 5’ untranslated region (UTR) that harbors an internal promoter, two open reading frames (ORF1 and ORF2; not drawn to scale), and a 3’ UTR that ends in a poly (A) tail. ORF2 contains an endonuclease (EN) and reverse transcriptase (RT) domains, as well as a cystiyne-rich 3’ end (C). The EGFP retrotransposition indicator cassette consists of a backward copy of the *EGFP* gene whose expression is controlled by the human cytomegalovirus major immediate early promoter (pCMV) and the herpes simplex virus thymidine kinase polyadenylation sequence (pA). This arrangement ensures that EGFP expression will only become activated upon L1 retrotransposition*.* The black arrows indicate PCR primers flanking the intron present in the *EGFP* gene. **b**, Bright field and UV images are shown of clone C6+ cells containing an L1 retrotransposition event derived from a single cell. Bar = 20 mm. **c**, PCR analysis of genomic DNA isolated from the clone C6+. PCR was conducted using the primers shown in panel **a**. The 1243-bp PCR product corresponds to the original L1 vector harboring the intron-containing *EGFP* indicator cassette. The 343-bp PCR product, diagnostic for the loss of the intron, is indicative of a retrotransposition event. Sequencing of the 343-bp PCR product confirmed the precise splicing of the intron (data not shown).

Previously characterized L1 clones in NPCs showed a neuronal differentiation tendency using a mixed differentiation protocol (Muotri et al., 2005). Then, we next examined whether the clone C6+ also has a similar neuronal predisposition. Interestingly, the ability of this clone to differentiate in neurons is 1,6 times higher compared to the original bulk HCN cells. Moreover, such neuronal tendency is gained mainly at the expense of glial cells since the number of astrocytes in the clone C6+ is reduced by 50% after differentiation (Figure 2).

**Figure 2 -:**
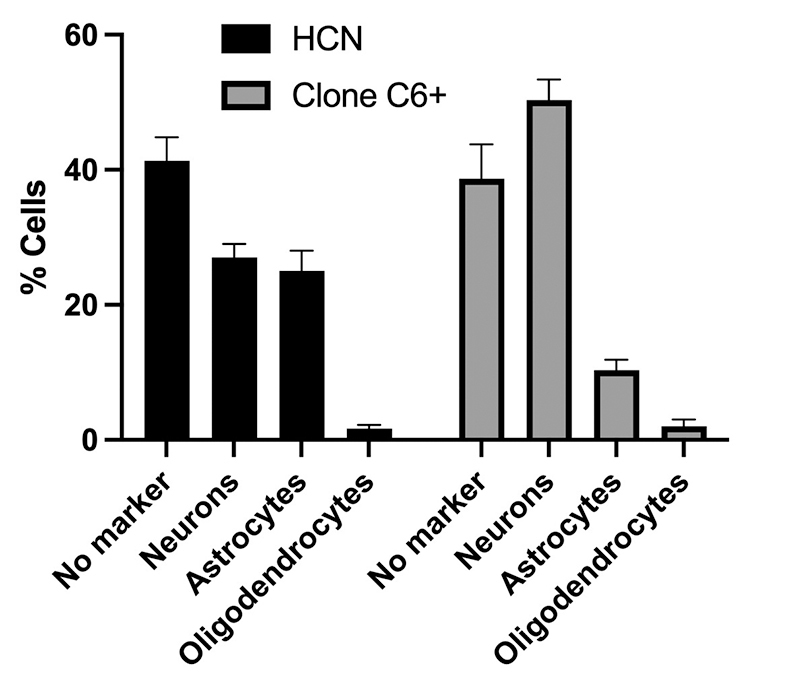
Neural differentiation for the clone C6+. After a mixed differentiation protocol using retinoic acid and serum (see Supplementary Material file Methods), cells were stained and quantified for neuronal (Map2ab), astrocytes (GFAP), or oligodendrocytes (RIP) markers. These experiments were performed in triplicates (n=3) and the bars represent standard deviation.

Inverse PCR was used to characterize the post-integration site from clone C6+ (Morrish et al., 2002). The insertion occurred at a preferred L1 EN consensus cleavage site (5’ - TTT/A - 3’) located on chromosome Xq22. This region is flanked by a small region on chromosome 10, suggesting a putative translocation between chromosome X and 10 (t(x;10)) upon L1 retrotransposition. The insertion site has a small TSD and a long polyA. The L1-EGFP element was ORF2 truncated at 6,176 bp (Figure 3). PCR analysis at this site to detect if the inversion was previously present in the parental HCN cells had proven difficult because of the presence of repetitive sequences nearby. Alternatively, we used rat probes for chromosome X in both HCN and clone C6+ mitotic chromosome spreads. In HCN cells, the FISH (fluorescence *in situ* hybridization) reaction revealed the presence of two signals in two independent chromosomes in HCN cells, suggesting the presence of two X chromosomes, as expected for a female-derived cell line. However, clone C6+ cells displayed an extra signal in a third chromosome (Figure 4). Finally, we use SKY (Spectral Karyotype) to confirm chromosome 10 as the other chromosomal arm in the putative translocation (Figure 4). Interestingly, the SKY also revealed that some cells from the C6+ clone also carry additional non-clonal chromosome aberrations, such as t(12;7) and t(15;6), which displays the potential for this cell line to change after long periods in culture. Such instability was not observed in the parental HCN cells, suggesting that chromosomal instability appears only in NPCs but not in neural stem cells.

**Figure 3 -:**
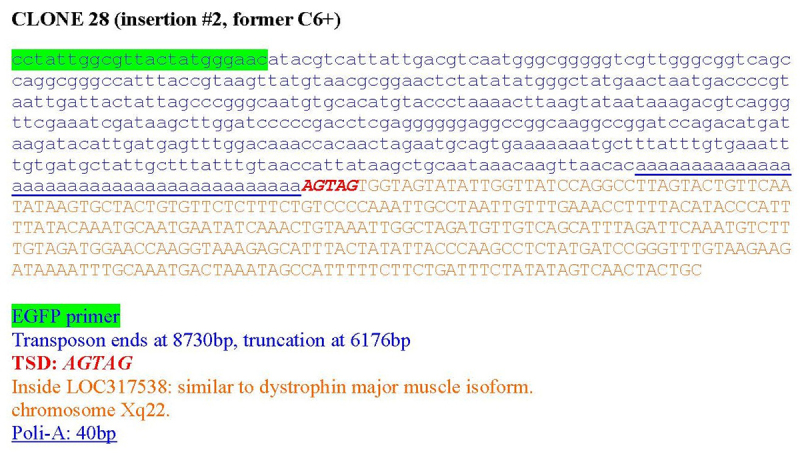
Schematic representation of the Clone 6+ L1 insertion. Inverse PCR indicated that the L1 was inserted in the antisense orientation of the dystrophin major muscle isoform (chromosome Xq).

**Figure 4 -:**
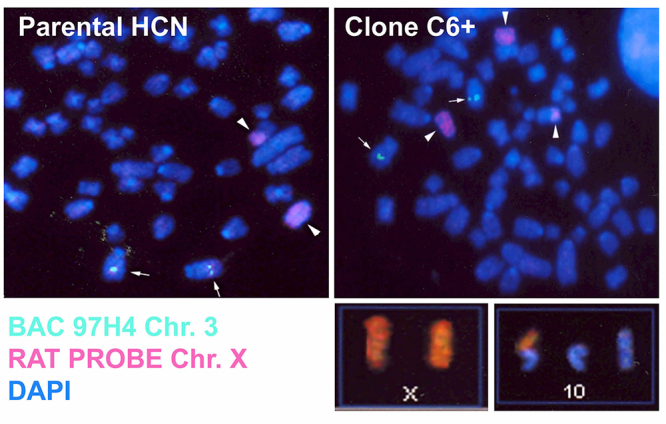
Inverse PCR putative interchromosomal translocation confirmation in Clone C6+ cells. Isolated cellular chromosomal spread in metaphase hybridized with X paint probe (arrowheads) and a BAC probe control for chromosome 3 (arrows) illustrates the extra fluorescence signal observed in the Clone C6+ but absence in the HCN parental cell. Bottom: spectral analysis of chromosomal spreads in the Clone C6+ revealed a translocation between chromosome X (brown) and chromosome 10 (blue).

Chromosomal translocations caused by L1 retrotransposons were proposed as a mechanism to explain the evolution of recent rodent L1 subfamilies and the occurrence of promoters swapping for non-LTR retroelements in certain avian species (Hayward et al., 1997; Saxton and Martin, 1998; Haas et al., 2001). A *de novo* L1 insertion using a similar system described here was previously described to map a putative interchromosomal translocation in HeLa cells (Gilbert et al., 2005). The fact that we observed a similar L1 insertion in HeLa and NPCs suggests that the intracellular environment requirements for interchromosomal translocation are present in both cell types. Together with this previous observation, we believe interchromosomal translocation can be regarded as a new genetic strategy by which L1 retrotransposons can shuffle DNA to new genomic locations.

The L1 target in the clone C6+ was inside the Loc317538 in chromosome Xq22, which is a region like the dystrophin major muscle isoform. Alterations in gene expression in this region do not seem to cause the strong neurogenic effect observed in this clone. However, the SKY experiment also revealed other, less frequent chromosomal translocations that may contribute to the final phenotype. While our data was observed using an ectopic L1 reporter construct, it is currently unknown if these other interchromosomal translocations were caused by endogenous L1 retrotransposition.

A chromosomal inversion involving parental L1 retrotransposon polymorphism has been correlated to autism in siblings (Vincent et al., 2006). At a somatic level, early reports applying FISH using individual chromosomal probes in postmitotic neurons revealed an extra signal, additional from two fluorescence spots expected for diploid cells, both in normal and Alzheimer’s brain tissues (Yang et al., 2001). Two interpretations for this phenomenon were proposed: (i) the extra signal could be related to a possible cell-cycle re-entering process associated with neuronal death, which would be more frequent in the disease brain (Herrup and Yang, 2007; Yang and Herrup, 2007; Pandey and Vinod, 2022; Yuen et al., 2022; Wong and Chow, 2022), or (ii) the extra signal may represent a gain of a chromosome in a constitutional and physiological aneuploidy process generated during brain development (Rehen et al., 2001, 2005; Arendt, 2012; Bajic et al., 2015). Based on our experimental data, we would like to propose a third, non-exclusive interpretation: the extra signal revealed by FISH in postmitotic neurons may be a consequence of a genetic rearrangement, such as interchromosomal translocation or duplication, caused by L1 retrotransposition in NPCs enrolled in neuronal differentiation. Our hypothesis agrees with the view that terminally differentiated cells, such as neurons, accommodate situations of genetic instability to dispense with the energy cost of maintaining global DNA repair systems (Nouspikel and Hanawalt, 2000, 2002, 2003).

## Supplementary material

The following online material is available for this article:

Methods.
