# Supplementary material for: Interchromosomal translocation in neural progenitor cells exposed to L1 retrotransposition
Source: Genet Mol Biol. 2023 Jan 30;46(1):e20220268. doi: 10.1590/1678-4685-GMB-2022-0268 (PMC9936793; doi:10.1590/1678-4685-GMB-2022-0268)
Supplement: Methods. [file 1415-4757-GMB-46-1-e20220268-s1.pdf]

Genetics and Molecular Biology
https://doi.org/10.1590/1678-4685-GMB-2022-0268

Supplementary Material to "Interchromosomal translocation in neural progenitor cells exposed to L1 retrotransposition"

Materials and Methods

Cell culture and immunofluorescence

HCN-A94 cells were isolated, characterized and cultured as described(Gage et al., 1995b; Palmer et al., 1997). For mixed pan neural differentiation, cells were cultured in N2 medium (Invitrogen) containing retinoic acid (RA, 1 μM, Sigma) and 0.1% FBS (Invitrogen) for 4 days before fixing(Muotri *et al.*, 2005). Immunofluorescence and cell quantification was performed as previously described(Gage et al., 1995a; Muotri *et al.*, 2005).

Fluorescence *in situ* hybridization (FISH) and SKY

Standard protocols for chromosome spread preparation in metaphase were used for FISH and counterstaining with DAPI (4',6-diamidino-2-phenylindole). DNA probe for chromosome 3 (BAC clone 97H4) was biotinylated with dATP(Heng et al., 1992). Rat chromosome X paint probe was purchased from Cambio. The procedure for FISH detection was performed according to standard protocols(Heng *et al.*, 1992; Heng and Tsui, 1993). Briefly, slides were baked at 55ºC for 1h. After RNase A treatment, the slides were denatured in 70% formamide in 2XSSC for 2 minutes at 70ºC, followed by dehydration with ethanol. Probes were denatured at 75ºC for 5 minutes in hybridization mix consisting of 50% formamide and 10% dextran sulphate. Probes were loaded on the denatured slides. After overnight hybridization, slides were washed and detected as well as amplified using a published method(Heng *et al.*, 1992). FISH signals were observed under fluorescent microcopy. Images were captures by CCD camera and merged

by RS Image software. For SKY, FISHed slides were used, following a standard protocol and chromosomes were karyotyped from 10 mitotic figures using a software developed by Applied Spectral Imaging(Heng et al., 2003).

Retrotransposition assay

Antibiotic selection (puromycin, 1 mg ml^{-1}) was begun 48h after electroporation. After 7 days, transfected puromycin-resistant cells were analyzed with a Becton Dickenson FACStar Plus containing a blue argon laser (488 nm) and fluorescein filter sets (530/30 bandpass). The EGFP PCR primers used here were previously described(Ostertag *et al.*, 2000). The L1 retrotransposition cassettes were gifts of Dr. H. H. Kazazian, Jr., Philadelphia, PA, USA.

Inverse PCR

Genomic DNA from the clone C6+ was digested for with *Ssp* I or *Xba* I, extracted with phenol and then chloroform, and subjected to overnight ligation. The products then were re-extracted, ethanol-precipitated and subjected to first round PCR amplification using the primers for the *EGFP* expression cassette. The L1 pre-integration sequence was identified using Blast (http://www.ncbi.nlm.nih.gov/BLAST/).
